# Donor Experience and Satisfaction: A Cross‐Sectional Survey of Australian Milk Donors

**DOI:** 10.1111/mcn.70169

**Published:** 2026-02-12

**Authors:** Claire Newman, Melissa K. Hyde, Abigail R.‐A. Edwards, Vanessa Clifford, Barbara M. Masser, Laura D. Klein

**Affiliations:** ^1^ Australian Red Cross Lifeblood Melbourne Victoria Australia; ^2^ School of Psychology The University of Queensland Brisbane Queensland Australia; ^3^ Royal Children's Hospital Parkville Victoria Australia; ^4^ Murdoch Children's Research Institute Parkville Victoria Australia; ^5^ University of Melbourne Parkville Victoria Australia; ^6^ National Institute for Health and Care Research Blood and Transplant Research Unit in Donor Health and Behaviour, Department of Public Health and Primary Care University of Cambridge Cambridge England UK

**Keywords:** breastfeeding, donor milk, human, human milk banks, lactation, milk, personal satisfaction, survey

## Abstract

Pasteurised donor human milk is a vital resource for vulnerable preterm infants. As demand continues to grow, the sustainability of milk banking services relies not only on recruiting donors but also on fostering positive donor experiences. Satisfied donors are more likely to contribute consistently and advocate for the service, enhancing its visibility and community support. A total of 588 formal milk bank donors who donated to Australian Red Cross Lifeblood milk programme between January 2024 and April 2025 were invited to participate in a survey. The survey aimed to explore factors that make milk donors feel valued, evaluate their satisfaction with different aspects of the donation process, and identify barriers that may hinder continued donation. The survey included Likert‐scale questions and optional open‐text responses. The survey was completed by 257 donors (43.7% response rate). Most (72.4%) felt highly valued by Lifeblood, especially due to receiving milk bags, and having supportive, personal interactions with donor coordinators. However, some donors felt undervalued due to a lack of post‐donation communication. Whilst satisfaction with the donation process was high, some respondents found the screening process repetitive and time‐consuming. The most reported barrier was limited freezer space (67.7%), followed by time constraints, illness in the household, and the burden of cleaning and sterilising equipment for milk expression at home. Milk banks should adopt donor‐centred practices, including streamlining processes to minimise time and effort required for donors, reduce costs incurred by donors where feasible, and enhance post‐donation transparency and engagement.

## Summary

- Supportive and respectful interactions with milk bank staff foster donor engagement by making donors feel valued and appreciated.
- Timely communication and recognition, such as updates and acknowledgements, enhance donor satisfaction and strengthen their connection to the milk bank.
- Reducing practical and financial barriers for milk donors, including simplifying screening, offering home collection, and providing milk storage supplies, makes donation more accessible and sustainable.

## 1 Introduction

Pasteurised donor human milk (PDHM) plays a vital role in reducing neonatal morbidity when maternal milk is unavailable or in short supply. This is particularly the case in very preterm infants who are at risk of necrotising enterocolitis (Quigley et al. 2024). As evidence supporting the use of PDHM for very preterm infants continues to grow, hospitals are experiencing a steady rise in demand (Shenker et al. 2023). This increasing need highlights the urgency of securing a reliable and sufficient supply of donations to maintain the sustainability of this essential service.

As with other Substances of Human Origin (SoHO), voluntary donors are the preferred source of PDHM. There are commonalities between the motivations for SoHo donors (Hyde et al. 2024) and the challenges services collecting SoHO face in meeting growing demand. For example, recruiting new donors represents a significant cost and time effort for all collection agencies. Donor retention, or encouraging recruited donors to make multiple donations, is one strategy to promote sustainable SoHO services. In the context of blood donation, a higher percentage of regular donors compared to first‐time donors is considered a marker of successful retention (Van Dongen 2015). For human milk, women who continue to donate milk over time tend to contribute larger volumes of milk compared to newly recruited donors (Kaech et al. 2022), so having engaged donors who contribute regularly supports milk banks to meet growing demand. However, unlike blood donors, who may remain eligible to donate for many years, milk donors face a limited window of eligibility due to the duration of lactation, milk bank requirements (which typically restrict donors to having infants under 12 months old) and return‐to‐work commitments that can impact ongoing breastfeeding and milk expression. Further, milk banks must carefully balance encouraging donation with ensuring that women do not donate milk at the expense of their own or their baby's health, and avoid any risk that their infants are not optimally breastfed (Israel‐Ballard et al. 2019).

Research has explored the experiences of SoHO donors, particularly blood donors, and has shown that blood donors are less likely to return if the process is perceived as time‐consuming or inconvenient (Custer et al. 2011; Van Dongen 2015). In the context of milk donation, systemic barriers have been highlighted in recent reviews and include receiving inadequate support from healthcare professionals, dissatisfaction with the donation process, and miscommunication with milk bank staff (Doshmangir et al. 2019; Mathias et al. 2023; Monti et al. 2023). For example, a US survey of 50 donors to non‐profit milk banks identified that the experience of complex rules, minimum donation volume requirements, and temporary exclusions due to illness or medication were barriers to continued donation (Wambach et al. 2019). Other US‐ and UK‐based surveys of milk donors reported screening processes as barriers to donation (Dos Santos et al. 2024; Rojjanasrirat et al. 2023). The need to streamline the donation process to reduce procedural challenges and thus promote willingness to donate and improve milk donor retention has been highlighted (Monti et al. 2023).

Having engaged milk donors who are willing to make repeated donations, and/or speak positively about their experiences with the service, will promote service sustainability (Kaech et al. 2022). Donor management practices, therefore, need to go beyond recruitment and focus on retention by fostering positive donor experiences, valuing donor contributions, and minimising barriers to donation. A recent qualitative study of Australian milk donors highlighted diversity in their experiences from feeling enabled and encouraged by their positive experiences to their ability to donate being hindered by process and personal barriers (Newman et al. 2025). There remains a significant gap, however, in understanding milk donors' experiences, particularly in the Australian context and in samples larger than those included in qualitative studies. Further, just over half (54%) of those who donate to Lifeblood, the primary non‐profit milk bank operator in Australia, only donate once (Klein et al. 2025); suggesting a need to explore the local factors influencing donor retention in milk banking in more depth.

To address these gaps, an exploratory study was undertaken with the following objectives to:

1. Examine what makes milk donors feel valued;
2. Assess the extent to which milk donors are satisfied with various aspects of the donation process; and
3. Identify barriers experienced by donors that may affect their ability to continue donating.

## 2 Methods

### 2.1 Study Design

A cross‐sectional survey of Australian Red Cross Lifeblood (Lifeblood) milk donors was undertaken. This study was reported in accordance with the Strengthening the Reporting of Observational Studies in Epidemiology (STROBE) guidelines for cross‐sectional studies.

### 2.2 Setting

Lifeblood currently supplies PDHM to hospitals across Australia. Lifeblood began collecting milk from donors in major metropolitan areas in two states (New South Wales and South Australia) in 2018, expanding to collect milk from donors in two additional states (Queensland and Victoria) in 2021 and 2024, respectively. Since 2018, around 2200 individuals have donated milk to Lifeblood. Donor recruitment involves an initial high‐level screening conducted over the phone. Eligible donors then receive a home visit for further assessment, which includes phlebotomy testing, and to obtain informed consent. Milk donations are collected during these home visits, with donor screening repeated at each collection to ensure ongoing eligibility.

### 2.3 Participants and Recruitment

Individuals who donated milk to Lifeblood between January 2024 and April 2025 (*n* = 588) were invited to complete the survey. Eligibility criteria for milk donation include meeting health and safety requirements. Donations are accepted from both mothers with a live infant and bereaved mothers. The survey did not collect specific data on whether donors had a live birth; therefore, the sample may include both groups. Donors received a text message containing an invite to participate and a link to access participant information and an electronic survey. A flyer containing a QR code to access the participant information and survey was also provided to donors via the donor coordinators during milk collection visits in March and April 2025. Respondents accessing the survey provided electronic consent and were able to opt‐in to win a gift voucher in appreciation of their time to complete the survey.

### 2.4 Data Collection

Survey questions were informed by a review of published literature on milk bank donor experiences and themes identified in a prior qualitative study of Lifeblood donors and potential donors (Newman et al. 2025). The draft survey was then piloted internally with researchers and milk operations staff to confirm clarity, appropriateness of wording, and alignment with study objectives. The survey questions were responded to on a 4‐point Likert scale, and related to: the extent experiences with Lifeblood made the donor feel valued (8 questions); satisfaction with information provided (12 questions); satisfaction with milk donation processes (11 questions); and extent to which barriers to donation were experienced (9 questions). Demographic information (8 questions) was also collected. Further, respondents were provided with the opportunity to type additional information at the end of each survey section. The survey was available in English only. Data were collected using REDCap (v14.0.30) survey platform. The survey remained open for 4 weeks.

### 2.5 Data Analysis

Survey entries were removed if they contained no data (*n* = 1) or incomplete data (*n* = 1; demographic questions answered only). Data were analysed using IBM SPSS statistical package, version 23. Descriptive statistics (number, percentage) were calculated for Likert scale questions and demographic information. Mean and standard deviation were also calculated for respondent age. To explore associations between respondent age and experience of barriers to donation, Spearman's Rho correlation coefficients were calculated. For categorical comparisons between respondent characteristics (first‐time donor vs. repeat donor, first‐time parent vs. more than one child, English primary language vs. other primary language, and history of blood donation vs. no history of blood donation) and reported barriers (response: not at all/unsure vs. a little/lot), a chi‐square test of independence with continuity correction was used. Statistical significance for these analyses was defined as *p* < 0.05. Chi‐square analyses were not conducted for questions relating to satisfaction with the donation process and information provided because the assumption of minimum expected cell counts was violated. Narrative data were subject to descriptive content analysis.

### 2.6 Ethical Approval

Ethical approval to conduct the study was received from Lifeblood Human Research Ethics Committee (2024#18‐LNR) and The University of Queensland Human Research Ethics Committee (2024/HE000885).

## 3 Results

### 3.1 Participant Demographics

A total of 257 donors completed the survey, representing a 43.7% response rate. The mean age of respondents was 32.6 years (SD: 4.49, range 24–43 years). Respondent demographic data is provided in Table 1. Most respondents resided in New South Wales (*n* = 116, 45.1%) followed by Queensland (*n* = 85, 33.1%). Two respondents lived in the Northern Territory at the time of data collection, donating whilst temporarily in a Lifeblood collection region.

**Table 1:** Respondent demographic data.

Descriptor	Respondents
	*n* (%)
State	
New South Wales	116 (45.1)
Queensland	85 (33.1)
South Australia	46 (17.9)
Victoria	7 (2.7)
Northern Territory	2 (0.8)
Undisclosed	1 (0.4)
Gender	
Woman/female	256 (99.6)
Non‐binary or gender diverse	1 (0.4)
Primary language	
English	208 (80.9)
Other	49 (19.1)
First‐time parent	
Yes	147 (57.2)
No	108 (42)
Undisclosed	2 (0.8)
Number of donations	
One time	114 (44.4)
Two times	69 (26.8)
Three or more times	73 (28.4)
Undisclosed	1 (0.4)
History of donating blood/plasma	
Yes	97 (37.7)
No	156 (60.7)
Undisclosed	4 (1.6)
Last blood/plasma donation (years)	
Less than two	14 (14.4)
Two to five	31 (40)
More than five	34 (35.1)
Undisclosed	18 (18.6)

Most respondents identified as female (*n* = 256, 99.6%) with English the primary language spoken at home (*n* = 208, 80.9%). Other languages spoken at home included Indonesian (*n* = 7, 2.7%), Mandarin (*n* = 5, 1.9%), Chinese (*n* = 5, 1.9%) and Vietnamese (*n* = 5, 1.9%). More than half of respondents were first‐time parents (*n* = 147, 57.2%) and most had donated milk two or more times (*n* = 142, 55.2%). Just over one‐third (*n* = 97, 37.7%) had previously donated blood or plasma, with the majority having done so more than 5 years ago (*n* = 34, 35.1%).

### 3.2 Feeling Valued as a Milk Donor

**Figure 1:**
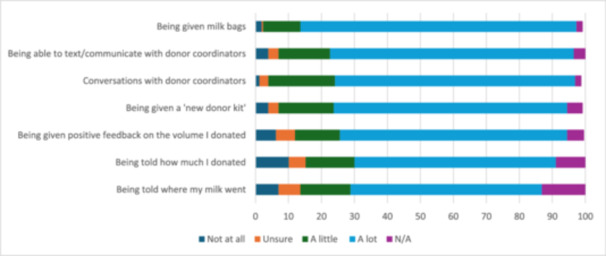
Aspects of donation that made donors feel valued (percentage).

Most respondents (*n* = 186, 72.4%) indicated that they felt highly valued as a donor by Lifeblood (Figure 1; see Appendix I for Likert scale responses). Only 2 respondents (0.8%) indicated that they did not feel valued at all. Aspects of donating most likely to make respondents feel highly valued were “being given milk bags” (*n* = 215, 83.7%), “being able to text/communicate with donor coordinators” (*n* = 190, 73.9%) and having “conversations with donor coordinators” (*n* = 187, 72.8%; Figure 1). Respondents most frequently reported that “being told how much they donated” or “where their milk went” did not occur (*n* = 23, 8.9% and *n* = 34, 13.2%, respectively). These were also the two factors most commonly identified as not making donors feel valued at all (*n* = 26, 10.1% and *n* = 18, 7%, respectively).

Eighty (31.1%) respondents provided 134 comments regarding further information about other aspects that made them feel valued by Lifeblood (or not). Most comments (*n* = 117, 87.3% of 134) related to aspects that made respondents feel valued as a donor and included having positive interactions with staff, being known by staff, feeling supported by staff throughout the process, and having a donation process that was easy and efficient:

The coordinator was always positive and supportive. Donating milk and having a little conversation with her made me feel I was contributing something during the time when I felt lonely staying at home with my baby.

When I called about donating at some point in the process, it was clear the staff recognised/knew my name and knew me as an individual. I valued the staff coming to my home throughout the process, making it as easy as possible for me to donate.

Other comments (*n* = 42, 31.3% of 134) related to aspects of milk donation that did not make respondents feel valued and included a lack of communication in relation to not being told whether their milk was used, where it was used, or how much they donated:

I didn't really feel valued at all, after my 1st donation the only contact I got was someone asking me to donate again, no info on how much was given or where it went either time.

I was told that I'd be informed about where my milk went but I never was, which was really disappointing.

### 3.3 Satisfaction With Information Provided

**Figure 2:**
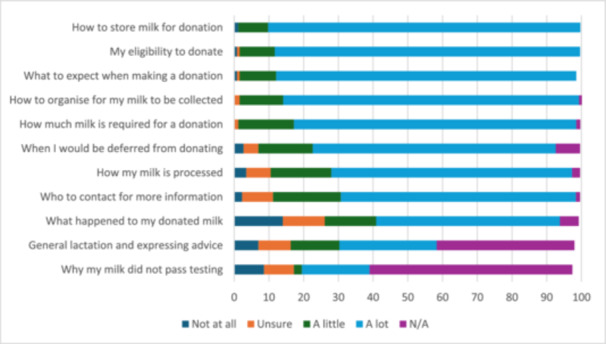
Satisfaction with information provided (percentage).

Regarding satisfaction with the provision of information (Figure 2; see Appendix I for Likert scale responses), respondents were most satisfied with the information received about: how to store milk for donation (*n* = 231, 89.9%), their eligibility to donate (*n* = 226, 87.9%), what to expect when making a donation (*n* = 222, 86.4%), and how to organise for their milk to be collected (*n* = 219, 85.2%). Respondents were most dissatisfied with the information provided to them on what happened to their donated milk (*n* = 36, 14%). Whilst not applicable to all respondents, others were also unsatisfied with the information received in relation to why their milk did not pass testing (*n* = 22, 8.6%) and general lactating and expressing advice (*n* = 18, 7%).

Respondents whose primary language spoken at home was not English (*n* = 49, 19.1%) were asked whether receiving information in English only affected their understanding. Of these, three respondents (6.1% of 49) reported that their understanding was inhibited, 31 (63.3% of 49) reported no impact, and 15 (30.6% of 49) did not answer the question.

Eighty‐two (31.9%) respondents provided 116 comments that further explained their satisfaction or dissatisfaction with the information provided. Most comments (*n* = 77, 66.4% of 116) reflected dissatisfaction regarding what happened to their milk after donating; specifically, how donated milk is tested and processed, whether the milk they donated passed testing and/or was used, where their milk went, the amount they donated, and who were the recipients of milk donated:

Would be nice to know volume and if my milk was suitable for donation. If so, where they went to (e.g., premmies).

No word after blood test. No word on milk donation. No word on if it got donated or in the bin.

Other comments (*n* = 18, 15.5% of 116) highlighted dissatisfaction with information about aspects prior to collection of milk, such as eligibility, factors that exclude or defer donating, storing milk, and scheduling milk collection:

For most people, already with a stash, they can be excluded quite easily and it's disappointing. Perhaps a leaflet… with clear bullet points about what needs to happen to be eligible.

The conditions for deferral periods should be given beforehand to interested donors. Only after I had my stash ready, I was questioned about meds and illnesses and told that lots of my milk cannot be donated.

The remaining comments (*n* = 16, 13.8% of 116) related to respondent satisfaction with being provided information about the process of donating, most often through their interactions with staff:

I felt very well informed by the information provided to me when I registered (from website) and from my initial call, then additional information given to me by the coordinator that came to my house.

### 3.4 Satisfaction With the Process of Donating

**Figure 3:**
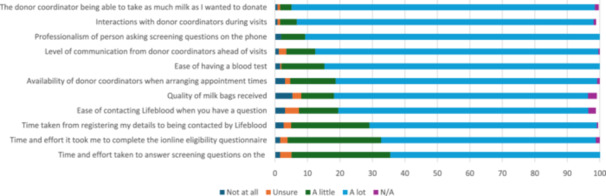
Satisfaction with the process of donating (percentage).

Regarding satisfaction with the process of donating (Figure 3; see Appendix I for Likert scale responses), respondents were most satisfied with donor coordinators being able to take as much milk as they wanted them to (*n* = 240, 93.4%). Respondents were also highly satisfied with processes that involved the donor coordinators including interactions during visits (*n* = 235, 91.4%), their professionalism when asking screening questions (*n* = 233, 90.7%), and their level of communication ahead of visits (*n* = 224, 87.2%). Whilst dissatisfaction was low across all processes, respondents were most dissatisfied with the quality of milk bags provided by Lifeblood (*n* = 14, 5.4%).

Forty‐eight respondents (18.7%) provided 59 comments that further explained their satisfaction or dissatisfaction with the process of donating. Most comments (*n* = 30, 50.8% of 59) related to respondent dissatisfaction, most often with the donation process (*n* = 22, 37.3% of 59), including length and inefficiency of the screening questionnaire, unsatisfactory milk bags, and concern about donated milk being wasted:

Admittedly completing the online questionnaire, followed by the phone questionnaire and finally the in‐person questionnaire is VERY tedious and time consuming.

It just broke my heart that what I considered at the time to be a large quantity of my milk was discarded. I understand that I agreed to the terms and conditions being if the milk doesn't pass for whatever reason it cannot be given back and needs to be disposed of however it was very hard to mentally absorb that.

Fewer comments (*n* = 8, 13.6% of 59) related to dissatisfaction with staff, including a lack of or delayed response from staff and feeling judged:

I texted 2 of the donor coordinators for pick up appointment but no one got back to me. Because I needed to clear up my freezer space, I ended up donating to a mom from a Facebook group.

I felt judged when I called to make the second donation. I was made to feel like I wasn't sanitising the equipment properly.

The remaining comments (*n* = 29, 49.2% of 59) related to respondent satisfaction with the process of donating, with these most often referring to the ease of the process, including having at home testing/collection, or having positive interactions with staff:

The coordinators were lovely and understanding. I did not feel judged even if I'm dishevelled or if my house was not as tidy as I'd like it to be. Communication prior to arriving for donation was good. I only considered (donating) because they were able to collect blood for screen at my house, which meant I did not have to go get a blood test done myself.

### 3.5 Barriers to Donating

**Figure 4:**
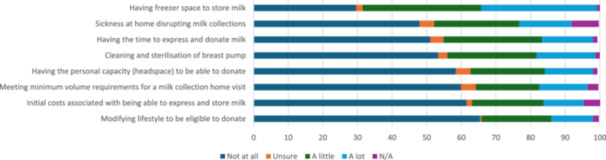
Barriers to donating milk (percentage).

The most common barrier to donating experienced either “a little” or “a lot” was having freezer space to store milk (*n* = 174, 67.7%; Figure 4; see Appendix I for Likert scale responses). Other barriers experienced either “a little” or “a lot” by respondents were time constraints (*n* = 111, 43.2%), sickness at home disrupting milk collections (*n* = 102, 39.7%) and cleaning/sterilisation of breast pump (*n* = 110, 42.8%).

A chi‐square test of independence was used to examine associations between respondent characteristics and reported barriers to donation. Respondents who had donated once only, or were first‐time parents, were more likely to report meeting the minimum volume requirements for a milk collection home visit as a barrier to donation, χ^*2*^ (1, *n* = 247) = 5.35, *p* = 0.021, Ф = −0.17 and *χ* ^*2*^ (1, *n* = 246) = 5.40, *p* = 0.020, Ф = −0.16, respectively. Respondents with more than one child were more likely to report sickness at home disrupting milk collections as a barrier to milk donation, *χ* ^*2*^ (1, *n* = 234) = 4.44, *p* = 0.035, Ф = 0.15.

Respondents who spoke a primary language other than English were more likely to report barriers to donating including: initial costs, χ^*2*^ (1, *n* = 245) = 22.0, *p* = 0.000, Ф = 0.31; having freezer space χ^*2*^ (1, *n* = 255) = 4.29, *p* = 0.038, Ф = 0.14; cleaning and sterilisation of equipment χ^*2*^ (1, *n* = 254) = 13.1, *p* = 0.001, Ф = 0.24; having personal capacity, χ^*2*^ (1, *n* = 252) = 8.51, *p* = 0.004, Ф = 0.19; having time to express and donate milk, χ^*2*^ (1, *n* = 252) = 7.29, *p* = 0.007, Ф = 0.18; and meeting minimum volume requirements χ^*2*^ (1, *n* = 248) = 5.76, *p* = 0.016, Ф = 0.16.

Respondent age was negatively correlated with initial costs associated with being able to express and store milk (*r* = −0.14, *n* = 243 *p* < 0.03), indicating younger donors are more likely to experience this as a barrier to donation. Key inferential statistics are presented in Table 2.

**Table 2:** Key inferential statistics.

	Test statistic	*n*	*p*	Effect size
Donated once				
Minimum volume requirement	*Χ* ^2^(1) = 5.35	247	0.021	–0.17
First‐time parent				
Minimum volume requirement	*Χ* ^2^(1) = 5.40	246	0.020	–0.16
Having more than one child				
Sickness at home disrupting collections	*Χ* ^2^(1) = 4.44	234	0.035	0.15
Non‐English primary language				
Initial costs	*Χ* ^2^(1) = 22.0	245	0.000	0.31
Freezer space	*Χ* ^2^(1) = 4.29	255	0.038	0.14
Cleaning and sterilisation of equipment	*Χ* ^2^(1) = 13.1	254	0.001	0.24
Personal capacity	*Χ* ^2^(1) = 8.51	252	0.004	0.19
Time	*Χ* ^2^(1) = 7.29	252	0.007	0.18
Minimum volume requirement	*Χ* ^2^(1) = 5.76	248	0.016	0.16
Age correlation				
Initial costs	*r* = –0.14	243	0.030	—

*Note:* All tests are two‐tailed.

Thirty‐nine respondents (15.2%) provided 49 comments that further explained the barriers they experienced when attempting to donate/donating milk. Most comments referred to being unaware of reasons for deferral/not understanding deferral periods (*n* = 13, 26.5% of 49), and unexpected costs experienced related to storage and purchase of milk bags (*n* = 13, 26.5% of 49):

I was not able to donate a decent volume of my milk due to taking (Ibuprofen) but not being able to confirm which dates… I wish they mentioned the limitation on Nurofen as I would have tracked my consumption or not taken it at all.

I had to worry about enough space (available) in the freezer to store the milk. I also had to get a new breast pump as my old one did not work which was costly.

Other comments indicated that meeting eligibility requirements (*n* = 7, 14.3% of 49) and a lack of information (*n* = 6, 12.2% of 49) were barriers to donation:

I understand why it's a minimum 3 L volume but it would be nice to accept a bit less too.

I think initially, getting the information on donating was daunting. It wasn't until a friend of mine explained the process that I was prompted to call. Being able to find eligibility info online and exactly how the storage/life span of the milk works would be very handy.

## 4 Discussion

Women who recently donated milk to Lifeblood generally felt valued and satisfied with their experience, particularly regarding the information provided and the ease of the donation process. For many, this positive experience was strongly influenced by warm, supportive, and respectful interactions with milk bank staff, highlighting their critical role in donor engagement and retention. Conceptually, this aligns with qualitative findings from Sweden, where some donors questioned whether they would donate again after experiencing unfriendly or unaccommodating interactions with milk bank staff (Olsson et al. 2021).

A positive donation experience is crucial for encouraging repeat donations. Donors who feel valued and recognised are more likely to return, a finding consistent with research on blood donors showing that pleasant donation experiences fosters positive attitudes and increase future donation likelihood (Van Dongen 2015). Similarly, qualitative studies indicate that encouragement and motivation from staff can influence continued participation (Machado et al. 2015; Newman et al. 2025). While supportive interactions are important for all SoHO donors, they may hold particular significance for milk donors, especially during periods of maternal role transition, when often navigating postpartum recovery, hormonal changes, or complex grief following infant loss, during a time when social support networks may be limited (Nowland et al. 2021). Providing both lactation and emotional support can enhance the donor experience and promote retention (Monti et al. 2023). Human resource challenges within milk banks have been linked to reduced volumes of milk collection (Mondkar et al. 2018), suggesting the need for investment in staff training and capacity to maintain a responsive and supportive service.

Our study also underscores the importance of post‐donation care and the need for clear communication about deferral periods. Service quality, including timely appreciation and feedback, fosters positive donor experiences (Martín‐Santana and Melián‐Alzola 2022), yet some respondents reported gaps in these areas. This may potentially stem from a mismatch between donor expectations, shaped by what they were told by the milk bank or by Lifeblood's reputation, and the follow‐up received. For instance, while blood donors in Australia receive prompt thank‐you messages and updates about where their donation went, Lifeblood milk donors typically receive only a thank‐you letter on their baby's first birthday. Given that most milk donations occur within the first 6 months, this delayed acknowledgement can leave donors feeling disconnected from the service. Participants expressed a desire for more timely and specific post‐donation feedback, particularly regarding the use of their donated milk and whether their serology and milk microbial testing results were acceptable. Currently, donors are only contacted if issues are identified in their serology or milk during testing. When a donors' milk does not meet microbial criteria on two or more occasions (excluding bereaved donors), they receive a follow‐up call to review cleaning practices for milk expression equipment. On average, approximately 7% of donations are discarded due to microbial results (Clifford et al. 2021). It is important to note that the discard rate reported by Clifford et al. (2021) reflects microbial screening outcomes at the Lifeblood milk bank, the same setting as the current study, and may not be generalisable to milk banks operating under different conditions, such as those sourcing exclusively from community donors or hospital‐based donors. Notably, nearly 9% of participants in this study reported dissatisfaction with information they received as to why their milk did not pass testing, suggesting a need for clearer and more proactive feedback. Further, providing reassurance when testing is passed could strengthen donor trust and reinforce appreciation for their contribution.

Consideration could also be given to alternative approaches for acknowledging and appreciating donors' contributions. For example, in Sweden, milk donors have even suggested that small, tangible tokens of appreciation, such as a badge, could help them feel acknowledged and allow them to share their contribution with others (Olsson et al. 2021). Given the limited timeframe for milk donation eligibility, timely appreciation and recognition are essential to encourage repeat donations and foster a positive donor experience.

Donors, in the current study, also reported dissatisfaction with the information provided around deferral periods. Many milk donors are highly motivated to avoid wasting milk (Candelaria et al. 2018; Wambach et al. 2019), and our results indicate unanticipated deferrals lead to frustration and a sense of lost milk, time and effort. Clear and early communication from the milk bank concerning the conditions that would result in deferral would enable donor preparation and reduce associated losses (Hyde et al. 2023). Qualitative research has highlighted that the time and energy required to donate milk, in general, are considerable (Olsson et al. 2021). Donors must undergo lengthy screening and serology testing, express milk regularly, clean and sterilise equipment, ensure proper storage, and coordinate with milk banks for collection; all while managing the demands of caring for an infant, other children, and in some cases, working. Recognising the time and energy associated with milk donation and having measures in place to avoid wastage of donor milk, time and energy is essential to improving donor satisfaction.

Our findings indicate that language may play a role in shaping donor experience and perceived barriers. While only a small proportion of respondents whose primary language was not English reported that receiving information in English inhibited their understanding, these donors were significantly more likely to report practical barriers such as initial costs, freezer space, cleaning and sterilisation, and time constraints. This suggests that language differences may intersect with other challenges, potentially amplifying barriers to donation. Barriers such as lack of information and logistical challenges may disproportionately affect milk donors with limited English proficiency (Chaudhari and Aiyer 2024; Mathias et al. 2023). Future research should explore the experiences of donors with limited English proficiency in greater depth, as those with severe language barriers were unlikely to have participated in this survey. Understanding these perspectives is critical for developing inclusive communication strategies and support systems that ensure equitable access to milk donation programmes.

To support retention, donation processes must be as easy and convenient as possible. Research on blood donors has shown that individuals are less likely to return if the process is perceived as time‐consuming or inconvenient (Custer et al. 2011; Van Dongen 2015). In the current study, survey respondents expressed dissatisfaction with the time and effort required for donor eligibility screening. Similarly, milk donors internationally have reported frustration with repetitive screening questions and stressful health checks (Monti et al. 2023), highlighting the need to simplify screening procedures where feasible. Any reduction in screening‐related procedures, however, must be carefully balanced against the imperative to ensure the safety of vulnerable infants receiving PDHM.

Consistent with findings from the current study, where participants are not required to travel to donate, home‐based milk collection has been identified as a key facilitator of continued donation. This approach helps overcome logistical and time‐related barriers, as supported by previous research (Kaech et al. 2022; Mathias et al. 2023; Olsson et al. 2021). International studies have also cited travel costs, time and logistics associated with milk transportation as significant obstacles to donation (Brown et al. 2025; Wambach et al. 2019), with potential donors in South Australia indicating they would be more likely to donate if the process were easier and less time‐consuming (Mackenzie et al. 2013).

Making milk donation as cost‐free as possible is likely to enhance donor satisfaction and retention through reducing financial barriers. Practical support, such as the provision of milk storage bags, was highly valued by donors in the current study. This aligns with findings from the US, where the cost of collection containers has been identified as a significant barrier to donation, and providing these items has been shown to ease the burden on donors (Rojjanasrirat et al. 2023; Wambach et al. 2019). Recent internal data from Lifeblood indicates that milk donors used an average of 94 milk storage bags during their donation period, with usage ranging from 3 to 1,910 bags. At an estimated cost of AUD$0.50 per bag, this equates to an average expense of AUD$47 per donor, excluding any bags associated with feeding their own infant. Another cost associated with milk donation is the purchase and maintenance of breast pumps, with around three‐quarters of European milk banks supplying them to donors for expression of milk to donate (Kontopodi et al. 2021) as a way to further alleviate the financial costs of milk donation.

The European Union regulation on SoHO (Regulation 2024/1938) reinforces the principle that unpaid donation should be financially neutral, meaning it should not result in either financial gain or loss for the donor (The European Parliament and of the Council 2024). While milk or any other SoHO donation is a voluntary and non‐remunerated act, and considered safest when it remains so, the regulation allows for both financial and non‐financial compensation to reimburse donors for expenses incurred or to offset any losses. Given the unique demands of milk donation, including costs not typically faced by blood or plasma donors, such as milk storage bags and freezer space, there is a compelling case for ensuring donors are adequately supported. Doing so not only prevents financial strain but also promotes continued participation in this vital service.

### 4.1 Methodological Strengths and Limitations

This study had several methodological strengths and limitations. A notable strength was the response rate, with participants representing more than 40% of all individuals who donated milk to Lifeblood in the previous 14 months. However, there is potential for sample bias, as those who were the most motivated or engaged with the service were likely more inclined to respond, while individuals who had disengaged may be underrepresented. The questionnaire used in this study was specifically developed for this research and, although informed by qualitative data, it was not a validated measure of milk donor satisfaction. Additionally, as a cross‐sectional study, it does not allow for examination of causal relationships or the impact of identified factors on donor retention over time. A longitudinal study would be required to explore these dynamics more comprehensively.

## 5 Conclusion

The donor experience is essential to maintaining donor engagement and fostering a sense of value and connection. This study highlights the critical role of milk bank staff in fostering donor satisfaction through respectful communication, emotional support, and timely feedback. To prevent disappointment at the point of first and subsequent donations, it is important to provide donors with clear and detailed eligibility criteria, particularly regarding medication use. Positive interactions not only affirm the donor's contribution but also build a sense of connection and trust in the service. Conversely, logistical challenges, unclear communication, and financial burdens can undermine the donor experience and may deter future participation.

To sustain and grow milk donation programmes, investment in donor‐centred practices is essential, including streamlining processes, reducing costs associated with milk donation for donors where feasible, and enhancing post‐donation engagement. Digital solutions, such as offering real‐time updates on donation status, personalised feedback on milk testing results, and mobile platforms for communication offer promising avenues to strengthen donor engagement. We are currently engaging with the Lifeblood milk programme to share these findings and explore opportunities for service improvement. Future research should evaluate the feasibility and impact of these interventions to ensure they meet donor needs effectively. By recognising and responding to the unique needs and motivations of milk donors, milk banks can cultivate lasting relationships that support both donor well‐being and sustainability of the service.

## Author Contributions

All authors contributed to the design and development of the project. C.N. and L.D.K. were responsible for data collection. C.N., M.K.H., and A.E. were responsible for data analysis. C.N. prepared the initial version of the manuscript, and all authors participated in editing and refining the final version. B.M., V.C. and L.D.K. contributed to receiving grants that funded this project.

## Conflicts of Interest

All authors are current employees or affiliates of Australian Red Cross Lifeblood, a not‐for‐profit organisation which provides pasteurised donor human milk from voluntary, non‐remunerated donors to Australian hospitals.

## Supporting information

Appendix I.

## Data Availability

Data supporting the results of this study are available on request from the research team pending approval from the Lifeblood Human Research Ethics Committee. Data are not publicly available due to privacy or ethical restrictions.
