# Supplementary material for: Donor Experience and Satisfaction: A Cross‐Sectional Survey of Australian Milk Donors
Source: Matern Child Nutr. 2026 Feb 12;22(1):e70169. doi: 10.1111/mcn.70169 (PMC12896371; doi:10.1111/mcn.70169)
Supplement: Supplementary file 1 — Appendix I. [file MCN-22-e70169-s001.docx]

# Appendix I

Likert scale responses associated with Figures 1-4

|  | **Not at all** | | **Unsure** | | **A little** | | **A lot** | | **N/A** | |
| --- | --- | --- | --- | --- | --- | --- | --- | --- | --- | --- |
|  | **n** | **%** | **n** | **%** | **n** | **%** | **n** | **%** | **n** | **%** |
| **Extent each donation aspect made you feel valued (Fig 1)** |  |  |  |  |  |  |  |  |  |  |
| Being told where my milk went | 18 | 7 | 17 | 6.6 | 39 | 15.2 | 149 | 58 | 34 | 13.2 |
| Being told how much I donated | 26 | 10.1 | 13 | 5.1 | 38 | 14.8 | 157 | 61.1 | 23 | 8.9 |
| Being given positive feedback on the volume I donated | 16 | 6.2 | 15 | 5.8 | 35 | 13.6 | 177 | 68.9 | 13 | 5.1 |
| Being given a 'new donor kit' | 10 | 3.9 | 8 | 3.1 | 43 | 16.7 | 182 | 70.8 | 12 | 4.7 |
| Conversations with donor coordinators | 3 | 1.2 | 7 | 2.7 | 52 | 20.2 | 187 | 72.8 | 5 | 1.9 |
| Being able to text/communicate with donor coordinators | 10 | 3.9 | 8 | 3.1 | 40 | 15.6 | 190 | 73.9 | 9 | 3.5 |
| Being given milk bags | 5 | 1.9 | 1 | 0.4 | 29 | 11.3 | 215 | 83.7 | 5 | 1.9 |
| **Satisfaction with the amount and quality of information (Fig 2)** |  |  |  |  |  |  |  |  |  |  |
| Why my milk did not pass testing | 22 | 8.6 | 22 | 8.6 | 6 | 2.3 | 50 | 19.5 | 150 | 58.4 |
| General lactation and expressing advice | 18 | 7 | 24 | 9.3 | 36 | 14 | 72 | 28 | 102 | 39.7 |
| What happened to my donated milk | 36 | 14 | 31 | 12.1 | 38 | 14.8 | 136 | 52.9 | 14 | 5.4 |
| Who to contact for more information | 6 | 2.3 | 23 | 8.9 | 50 | 19.5 | 174 | 67.7 | 3 | 1.2 |
| How my milk is processed | 9 | 3.5 | 18 | 7 | 45 | 17.5 | 178 | 69.3 | 6 | 2.3 |
| When I would be deferred from donating | 7 | 2.7 | 11 | 4.3 | 40 | 15.6 | 180 | 70 | 18 | 7 |
| How much milk is required for a donation | 0 | 0 | 3 | 1.2 | 41 | 16 | 209 | 81.3 | 3 | 1.2 |
| How to organise for my milk to be collected | 0 | 0 | 4 | 1.6 | 32 | 12.5 | 219 | 85.2 | 2 | 0.8 |
| What to expect when making a donation | 2 | 0.8 | 2 | 0.8 | 27 | 10.5 | 222 | 86.4 | 0 | 0 |
| My eligibility to donate | 2 | 0.8 | 2 | 0.8 | 26 | 10.1 | 226 | 87.9 | 0 | 0 |
| How to store milk for donation | 3 | 1.2 | 0 | 0 | 22 | 8.6 | 231 | 89.9 | 0 | 0 |
| **Satisfaction with the donation process (Fig 3)** |  |  |  |  |  |  |  |  |  |  |
| Time and effort taken to answer screening questions on the | 4 | 1.6 | 9 | 3.5 | 78 | 30.4 | 166 | 64.6 | 0 | 0 |
| Time and effort it took me to complete the online eligibility questionnaire | 4 | 1.6 | 6 | 2.3 | 74 | 28.8 | 170 | 66.1 | 3 | 1.2 |
| Time taken from registering my details to being contacted by Lifeblood | 7 | 2.7 | 6 | 2.3 | 62 | 24.1 | 180 | 70 | 1 | 0.4 |
| Ease of contacting Lifeblood when you have a question | 8 | 3.1 | 11 | 4.3 | 31 | 12.1 | 198 | 77 | 6 | 2.3 |
| Quality of milk bags received | 14 | 5.4 | 7 | 2.7 | 26 | 10.1 | 201 | 78.2 | 7 | 2.7 |
| Availability of donor coordinators when arranging appointment times | 8 | 3.1 | 4 | 1.6 | 36 | 14 | 207 | 80.5 | 2 | 0.8 |
| Ease of having a blood test | 4 | 1.6 | 1 | 0.4 | 34 | 13.2 | 218 | 84.8 | 0 | 0 |
| Level of communication from donor coordinators ahead of visits | 3 | 1.2 | 6 | 2.3 | 23 | 8.9 | 224 | 87.2 | 1 | 0.4 |
| Professionalism of person asking screening questions on the phone | 5 | 1.9 | 0 | 0 | 19 | 7.4 | 233 | 90.7 | 0 | 0 |
| Interactions with donor coordinators during visits | 2 | 0.8 | 2 | 0.8 | 13 | 5.1 | 235 | 91.4 | 2 | 0.8 |
| The donor coordinator being able to take as much milk as I wanted to donate | 2 | 0.8 | 2 | 0.8 | 9 | 3.5 | 240 | 93.4 | 3 | 1.2 |
| **Barriers to donating (Fig 4)** |  |  |  |  |  |  |  |  |  |  |
| Modifying lifestyle to be eligible to donate | 168 | 65.4 | 1 | 0.4 | 52 | 20.2 | 31 | 12.1 | 4 | 1.6 |
| Initial costs associated with being able to express and store milk | 158 | 61.5 | 4 | 1.6 | 53 | 20.6 | 30 | 11.7 | 12 | 4.7 |
| Meeting minimum volume requirements for a milk collection home visit | 154 | 59.9 | 11 | 4.3 | 47 | 18.3 | 36 | 14 | 8 | 3.1 |
| Having the personal capacity (headspace) to be able to donate | 150 | 58.4 | 11 | 4.3 | 55 | 21.4 | 36 | 14 | 3 | 1.2 |
| Cleaning and sterilisation of breast pump | 137 | 53.3 | 7 | 2.7 | 66 | 25.7 | 44 | 17.1 | 3 | 1.2 |
| Having the time to express and donate milk | 131 | 51 | 10 | 3.9 | 73 | 28.4 | 38 | 14.8 | 3 | 1.2 |
| Sickness at home disrupting milk collections | 123 | 47.9 | 11 | 4.3 | 63 | 24.5 | 39 | 15.2 | 20 | 7.8 |
| Having freezer space to store milk | 76 | 29.6 | 5 | 1.9 | 88 | 34.2 | 86 | 33.5 | 2 | 0.8 |
